# Expression patterns of five polymorphic membrane proteins during the *Chlamydia abortus* developmental cycle

**DOI:** 10.1016/j.vetmic.2012.06.017

**Published:** 2012-12-07

**Authors:** Nick Wheelhouse, Michelle Sait, Kim Wilson, Kevin Aitchison, Kevin McLean, David G.E. Smith, David Longbottom

**Affiliations:** a Moredun Research Institute, Pentlands Science Park, Bush Loan, Edinburgh, Midlothian EH26 0PZ, UK; b Institute of Infection, Immunity and Inflammation, College of Medical, Veterinary and Life Sciences, University of Glasgow, Garscule Estate, Bearsden Road, Glasgow G61 1QH, Scotland, UK

**Keywords:** *Chlamydia abortus*, Polymorphic membrane protein, Antigenic variation

## Abstract

It has been suggested that polymorphic membrane proteins (Pmps) belonging to the Type V autotransporter protein family play an important role in the pathogenesis of *Chlamydia abortus* (*C. abortus*; formerly *Chlamydophila abortus*) infection. In a previous study we demonstrated the expression of all the *pmps* at the transcriptional level. The purpose of this study was to measure the number of Pmp positive inclusions throughout the *C. abortus* developmental cycle to investigate heterogeneity in expression patterns. McCoy cells were infected with *C. abortus* and analysed for Pmp expression over a 72 h period by fluorescent immunocytochemistry. Pmp18D could be detected at all analysed time points, and could only be accurately quantified from 36 hpi while Pmp10G positive inclusions could be visualised from 36 hpi. Expression of Pmps 13G, 16G and 17G could only be visualised later in the cycle and within less than half of visualised inclusions. These results indicate that while expression of specific Pmps is constitutive (Pmp18D), the pattern of expression of other Pmps is more variable. This suggests that different members of the Pmp family may play different roles within the developmental cycle of the organism, with some (Pmps10G and 18D) having roles throughout the cycle, while the heterogeneity of expression of others may aid in antigenic variation.

## 1 Introduction

*Chlamydia abortus* (*C. abortus*: formerly *Chlamydophila abortus*) is the aetiological agent of ovine enzootic abortion (OEA), the single most common infectious cause of ovine abortion in the United Kingdom (Aitken and Longbottom, 2007). Like other members of the *Chlamydiaceae*, *C. abortus* is a Gram-negative, obligate intracellular pathogen and undergoes a biphasic developmental cycle. The infectious elementary body (EB) infects a host cell where it resides within a vacuole known as an inclusion, before rapidly undergoing conversion to a metabolically active form known as the reticulate body (RB). Between 48 and 72 h post-infection (hpi), the RBs re-condense to EBs and the inclusion and host cell are lysed at the end of the cycle to release the infectious organisms (Longbottom and Coulter, 2003).

*Chlamydia abortus* Pmps were originally identified through their immunogenicity with convalescent sheep serum (Longbottom et al., 1996). These proteins have been identified as autotransporters of the Type V secretion system (Henderson and Lam, 2001). However, uniquely the Pmps of all sequenced chlamydial species are additionally characterised by conserved domains consisting of an N-terminal repeat sequence (GG[A/L/V/I][I/L/V/Y] and FXXN) and Pmp-middle domain (Thomson et al., 2005). Pmp encoding genes have been identified in all sequenced members of the *Chlamydiaceae* but there is significant heterogeneity in gene carriage between species, with *C. trachomatis*, *C. pneumoniae*, *C. abortus*, *C. caviae* and *C. felis* genomes encoding 9, 21, 18, 17 and 20 *pmps*, respectively (Stephens et al., 1998; Kalman et al., 1999; Thomson et al., 2005; Read et al., 2003; Azuma et al., 2006).

The Pmps are of potential importance in understanding the virulence and pathogenesis of chlamydial infection and are important diagnostic and vaccine targets. The purpose of this study was to measure the number of Pmp positive inclusions throughout the *C. abortus* developmental cycle to determine any heterogeneity in expression patterns.

## 2 Materials and methods

### 2.1 Cell culture, propagation of *C. abortus* and cellular infection studies

The *C. abortus* strain S26/3 was propagated and titrated as described previously (Graham et al., 1995). To investigate the *in vitro* expression of Pmps in *C. abortus*, McCoy cells were grown to sub-confluence in 8-well chamber slides (BD Biosciences, Erembodegem, Belgium). Cells were infected with *C. abortus* at an estimated multiplicity of infection (MOI) of 1. Cells were fixed at 24, 36, 48, 60 and 72 hpi in ice-cold acetone, air-dried and stored at −20 °C until analysis.

### 2.2 Production of anti-Pmp polyclonal antibodies

Affinity purified rabbit polyclonal antibodies were produced commercially (Eurogentec, Southampton, UK). Antibodies were raised against specific peptides identified within the N-terminal passenger domain of Pmps 10G, 13G, 16G, 17G and 18D that were identified to be unique for each Pmp by BLAST analysis (Table 1) and validated for specificity by Western blot against each *C. abortus* recombinant Pmps (see Supplementary methods and Supplementary Fig. 1).

### 2.3 Fluorescent immunocytochemistry

Due to its high level of constitutive expression throughout the chlamydial developmental cycle, the Omp-1 protein was used to visualise the total number of infected cells using the anti-Omp-1 mAb 4/11 (Vretou et al., 1996). Binding of the rabbit anti-Pmp pAb was detected using a FITC conjugated anti-rabbit IgG secondary antibody (Sigma–Aldrich, Dorset, UK). An anti-mouse IgG Alexafluor^®^-597 nm secondary antibody was used for detection of Omp-1 mAb binding.

Slides were removed from −20 °C and rehydrated in PBS for 5 min before blocking in 2% BSA for 30 min at RT. The slides were incubated for a further 60 min with the appropriate antibody or pre-immune rabbit serum at RT. After washing 3 times in PBS, the chambers were removed and the slides were incubated with secondary antibodies for 30 min at RT in a light-tight humidity chamber, before washing a further 3 times in PBS and mounting using Prolong Gold^®^ anti-fade reagent containing DAPI (Invitrogen). Slides were examined using a digital imaging system with an Axioscope fluorescent microscope (Carl Zeiss Ltd., UK) equipped with GFP, PI and DAPI fluorescent filter sets and Cell* Imaging Software (Soft Imaging Systems, Münster, Germany) for image capture. Protein expression was quantified by determining the number of Pmp-positive inclusions expressed as a percentage of a total of 500 Omp-1 positive inclusions. Data were analysed by ANOVA (Genstat version 7 statistical package) using Fisher's least significant difference test to separate the means at both the 5% and 1% probability levels.

## 3 Results

### 3.1 Pmp protein expression

**Fig. 1:**
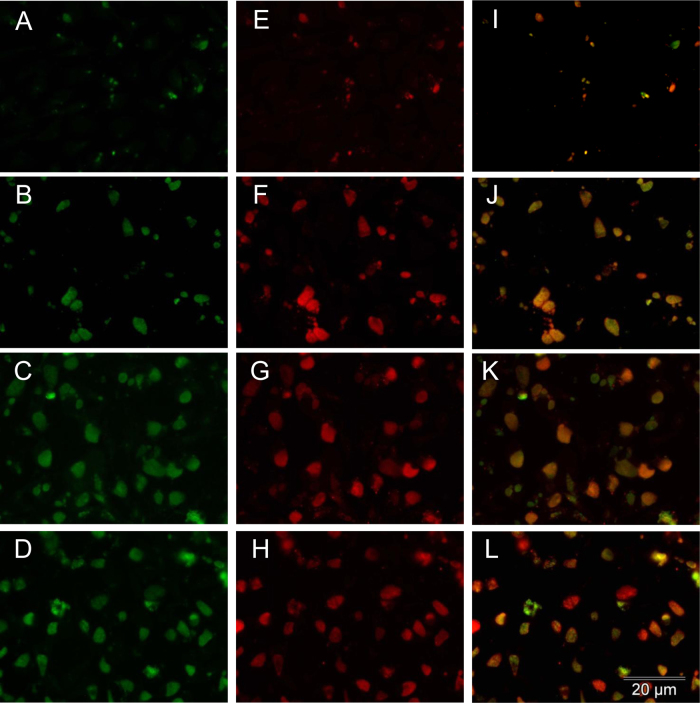
Fluorescent micrographs demonstrating the expression of Pmp18D throughout the developmental cycle of *C. abortus*. Fluorescent immunocytochemistry was carried out as described (Section 2). Slides were incubated with α-Pmp18D and α-Omp-1 antibodies and visualised with α-rabbit-FITC (green, panels A–D) and α-mouse-Alexafluor 598 (red, panels E–H) antibodies, respectively. Combined images are also shown (panels I–L) for time points: 36 h (A, E, and I), 48 h (B, F, and J), 60 h (C, G, and K) and 72 h (D, H, and L). All micrographs were obtained at the same magnification, and a scale bar is highlighted in panel (L).

**Fig. 2:**
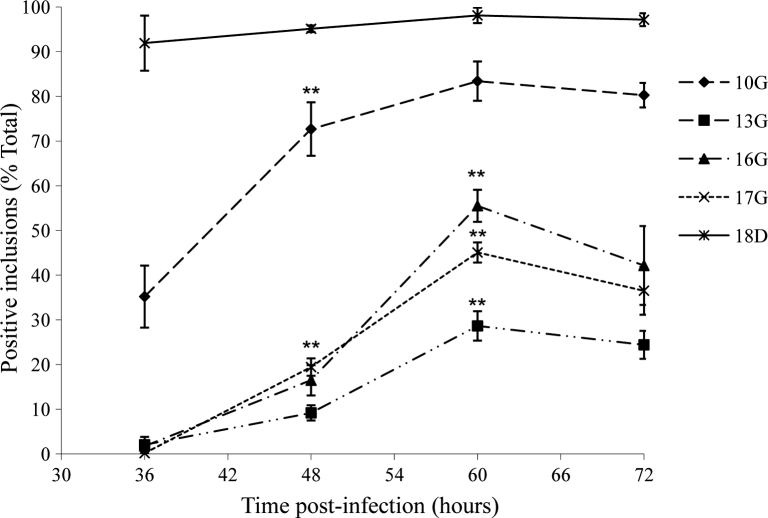
Proportion of inclusions expressing each Pmp at each time-point. Fluorescent immunocytochemistry was carried out as described (Section 2). The total number of inclusions at each time-point were visualised and counted using an α-Omp-1 antibody (visualised with α-mouse-Alexafluor 598). The number of Pmp-positive inclusions was counted using a specific α-Pmp polyclonal Ab (visualised with α-rabbit-FITC). Results were expressed as the mean proportion of Pmp to Omp-1 positive inclusions ± SEM of three independent experiments. *P < 0.05, **P < 0.01 mean difference between consecutive time-points.

The frequency of detection of Pmp positive inclusions was determined as a proportion of Omp-1-positive inclusions at each time-point. Expression of Pmp18D could be detected at all analysed time points, however due to the small size of the inclusions at 24 hpi could only be accurately quantified from 36 hpi (Fig. 1). At 36 h both Pmp18D and 10G expression could be observed in 91.9 ± 6.2% and 35.2 ± 6.9% of inclusions respectively (*P* < 0.01 above baseline). At 48 hpi, expression of Pmp18D remained elevated (95.1 ± 0.7%) and Pmp10G expression could be visualised in a significantly greater number of inclusions than at 36 hpi (72.7 ± 6.0%, *P* < 0.01). Conversely, the frequency of expression of Pmp16G (16.5 ± 3.4%, *P* < 0.01) and Pmp17G (19.4 ± 2.0%, *P* < 0.01) positive inclusions could only be determined in significant numbers from 48 hpi onwards and Pmp13G only from 60 h (28.6 ± 3.3%, *P* < 0.01). The number of Omp-1 positive inclusions expressing each of the analysed Pmps was maximal at 60 hpi (Fig. 2). However, while Pmp18D could be detected in nearly all Omp-1 positive inclusions (98.1 ± 1.7%) at 60 h, the other Pmps could only be detected in a proportion of the inclusions, 28.6 ± 3.3% for Pmp13G, 45.1 ± 2.2% for Pmp17G, 55.5 ± 3.6% for Pmp16G and 83.4 ± 4.4% for Pmp10G. Numbers of inclusions expressing each of the Pmps at 72 hpi were similar and not significantly different to those at 60 hpi (Fig. 2).

## 4 Discussion

Previously, variation in the timing and level of gene expression was observed for 15 *pmp* genes in *C. abortus* (Wheelhouse et al., 2009). From these results it was hypothesised that there would be considerable variation in the number of Pmp positive inclusions during a single infection with *C. abortus*, a process which could be significant in contributing towards antigenic variation. The Pmps analysed during the current study (Pmp10G, Pmp16G, Pmp17G, Pmp18D) were investigated as they were found to be expressed at a higher level than the other *pmp*s in the previous transcription study (Wheelhouse et al., 2009). The exception was Pmp13G, which was also included in the current study as one of the initially identified Pmps (Longbottom et al., 1998) and its similarity in structure to Pmp16G.

Inclusions expressing Pmp18D could be observed throughout the cycle even at the earliest time point of 24 h (although could not be accurately quantified). Experimental evidence has raised the suggestion that PmpD may contribute to the adhesion of *Chlamydia* to host cells, as antibodies raised against the N-terminal domain have been at least partially successful in inhibiting the *in vitro* infection of both *C. pneumoniae* (Wehrl et al., 2004) and *C. trachomatis* (Crane et al., 2006). However, in *C. trachomatis* PmpD, surface expression in the RB was greater than that found in the EB (Swanson et al., 2009) indicating a role for the protein during the metabolically active phase of the *C. trachomatis* developmental cycle. The observation of Pmp18D expression at even the earliest time-points during the current study would also suggest roles for this protein in the metabolically active phase of the *C. abortus* developmental cycle. The assignment of Pmps to different families was previously determined by alignments in the Pmp-M domain and carboxy-terminus beta-barrel sequences of Pmps from different species (Thomson et al., 2005). Amino acid similarity is higher among the barrel sequences however there is considerable sequence variation across passenger domains between *C. abortus* Pmp18D and *C. trachomatis* PmpD with 36% amino acid similarity by BLAST. However, while there is variation in amino acid sequence, there is a high level of conservation of crude protein structure and this does not preclude PmpD from performing similar functions across chlamydial species.

From the previous mRNA expression study, Pmp10G expression was observed throughout the developmental cycle (Wheelhouse et al., 2009) and the number of positive inclusions from 36 hpi is consistent with these findings. The function of this protein, as with the other Pmps remains elusive. However, given this almost constitutive pattern of expression it is likely that the protein is expressed in both the RB and EB and thus may perform different functions in the developmental cycle.

Consistent with our previous study investigating *pmp* transcript levels, where peak expression of the majority of the *pmp*s including *pmp13G*, *pmp16G* and *pmp17G* was observed at 60 hpi (Wheelhouse et al., 2009), the maximum number of inclusions expressing any of these Pmps was found to be at 60 hpi. However, even at this time-point the Pmps could only be visualised in a proportion of inclusions with 29%, 55% and 45% of inclusions positive for Pmp13G, 16G and 17G respectively. These proteins were originally identified through their immunogenicity (Pmp12/17G (Pomp90A/B), Pmp13G (Pomp91A) and Pmp16G (Pomp91B)) (Longbottom et al., 1998), however the rates of expression observed in the current study suggests the possibility of antigenic variation among a single population of *C. abortus*. After the initial identification of Pmp16G as an immunogenic protein the *pmp16G* gene was identified in the *C. abortus* S26/3 genome as a pseudogene due to the presence of a frame-shift in a centrally located polyguanine tract. However, during the sequence assembly coding sequences of varying length were identified (Thomson et al., 2005). This suggested potential phase-variation of the *pmp16G* gene, which has been observed for the *pmp* genes of other chlamydial species containing homopolymeric tracts (Pedersen et al., 2001) and that *pmp16G* is not a classical pseudogene but can be differentially expressed by the organism.

Heterogeneity in Pmp expression has been recently demonstrated in *C. trachomatis* (Carrasco et al., 2011; Tan et al., 2010), however, in contrast to *C. abortus*, the levels of variation were modest with expression of each of the Pmps in at least 90% of the inclusions quantified (Tan et al., 2010). These differences between *C. trachomatis* and *C. abortus* could at least be partially explained by the expansion in *pmp* gene carriage, particularly in the *pmp G/I* family: the *C. trachomatis* genome encodes 9 *pmp* genes in total (termed A–I) with a single *pmpG* and *pmpI* (Stephens et al., 1998), however the *C. abortus* genome encodes 18 *pmp* genes including 11 *pmpG* family genes (Thomson et al., 2005).

## 5 Conclusion

This study has demonstrated a high level of heterogeneity in the frequency of on and off switching of *pmp* gene expression in a population of cells infected with *C. abortus*, both in terms of timing and proportion of inclusions expressing each protein. The observation that Pmp10G and 18D are expressed earlier in the *C. abortus* developmental cycle compared to Pmp13G, 16G and 17G suggests a different role for these proteins. Additionally, the observation that some Pmps may be expressed by only a proportion of infected cells supports a role in antigenic variation.

## Competing interests

The authors declare that no competing interests exist that may have influenced the content of the manuscript.

## Figures and Tables

**Table 1:** Peptides used to generate polyclonal antibodies.

Protein	Immunising peptide
Pmp10G	DQTSSIKVQENVDIK
Pmp13G	SGSSGSITKPTTNLE
Pmp16G	ANTGGSTEIELNKTE
Pmp17G	EEKARAENLASTFN
Pmp18D	EKPIHAQGPKKGETD
