# Supplementary material for: Expression patterns of five polymorphic membrane proteins during the Chlamydia abortus developmental cycle
Source: Vet Microbiol. 2012 Dec 7;160(3-4):525–9. doi: 10.1016/j.vetmic.2012.06.017 (PMC3504296; doi:10.1016/j.vetmic.2012.06.017)
Supplement: Supplementary file 1 [file mmc1.docx]

**Supplementary methods**

Generation of anti-Pmp polyclonal antibodies and recombinant proteins for antibody validation

*Generation of recombinant Pmps*

To test the specificity of the antibodies, the N-terminal passenger domains of all the *C. abortus* Pmps were PCR-amplified (Supplementary Table 1), cloned and expressed. Passenger domains were identified using structural predictions resulting from use of the Yaspin hidden neural network secondary structure prediction program (Lin et al., 2005) to determine the presence of the α-helix linker at the start of the β-barrel domain (Henderson et al., 1998). Putative signal sequences were identified using PSORT (Nakai and Horton, 1999). The passenger domains, excluding the signal sequences, were amplified using the Expand Long-Template PCR kit (Roche Diagnostics, Burgess Hill, UK) and specific primers, which were designed using the Primer 3 program (Rozen and Skaletsky, 2000) (Table 2). PCR products were purified using the QIAquick^®^ gel extraction kit (Qiagen, Crawley, West Sussex, UK), cloned into the pET102/D-TOPO^®^ expression vector and transformed into One Shot^®^ Top10 competent *E. coli* (Invitrogen, Paisley, UK) and transformants selected on the basis of ampicillin resistance. Colonies were selected for overnight culture and plasmids were purified using the QIAprep^®^ spin miniprep kit (Qiagen). The presence of the insert and its orientation within the plasmid was verified by restriction digestion. The fidelity of the sequence of the cloned fragments was confirmed by dideoxy chain termination / cycle sequencing on an ABI 3730XL DNA sequencer, using sequencing primers corresponding to the T7 promoter and termination sites flanking the inserts (Eurofins MWG Operon, Ebersberg, Germany). Plasmids containing a correctly orientated insert were transformed into One Shot^®^ BL-21 Star^™^ (DE3) *E. coli* which were grown overnight in 10ml ampicillin-containing LB medium prior to induction with IPTG. After induction, the bacteria were pelleted by centrifugation for 10 minutes at 1800 x *g*. The medium was discarded and the bacterial cell pellets were resuspended in 1ml Bugbuster® master mix (Novagen^®^, Merck KGaA, Darmstadt, Germany). After digestion at room temperature for 20 mins the suspensions were centrifuged for 30 mins at 4°C and 14,000 x *g*. The supernatants were removed and retained in a fresh microcentrifuge tube for analysis and the insoluble pellets were resuspended in 1ml PBS by sonication. Protein samples (equivalent volumes of supernatant and resuspended pellets) were separated by SDS-PAGE and visualised using Simply-blue colloidal coomassie blue (Invitrogen). All passenger domain constructs were expressed as single recombinant proteins except for Pmps 1B, 15G and 18D. The passenger domains for each of these 3 proteins were expressed as either 2 (Pmp1B, 15G), or 3 overlapping fragments (Pmp18D) (Supplementary Table). Bands corresponding to the induced proteins were excised from the gel prior to trypsin digestion and subsequent MALDI-TOF analysis. Protein identification was confirmed by comparison of analysed peptide sequences against a MASCOT database ([www.matrixscience.com](http://www.matrixscience.com)) with subsequent identification of multiple specific peptide matches by BLASTp against the NCBI non-redundant protein database.

*Antibody cross-reactivity*

Affinity-purified antiserum raised against specific peptides were tested for their immunoreactivity and specificity by Western blotting against the validated recombinant Pmp constructs, using a HRP-conjugated anti-rabbit secondary antibody and 3,3'-Diaminobenzidine (SigmaFast™, Sigma-Aldrich, Dorset, UK) for visualisation. Each of the antibodies reacted with each of the relevant expressed Pmp constructs (Figure 1). A number of additional reactive bands of lower than expected molecular weight were identified by each antibody. Analysis of some of these bands by MALDI-TOF identified peptide sequences corresponding to each of the specific constructs and perhaps indicates products of protein degradation or processing. The specificities of each of the antibodies targeting unique peptides in Pmp10G, Pmp13G, Pmp16G, Pmp17G and Pmp18D were determined and tested for cross reactivity against each of the remaining recombinant Pmps. Each polyclonal antibody was specific for the relevant Pmp recombinant protein with no cross-reactivity observed (Supplementary Figure 1).

References

Henderson, I.R., Navarro-Garcia, F., Nataro, J.P., 1998. The great escape: structure and function of the autotransporter proteins. Trends Microbiol 6, 370-378.

Lin, K., Simossis, V.A., Taylor, W.R., Heringa, J., 2005. A simple and fast secondary structure prediction method using hidden neural networks. Bioinformatics. 21, 152-159.

Nakai, K., Horton, P., 1999. PSORT: a program for detecting sorting signals in proteins and predicting their subcellular localization. Trends Biochem. Sci. 24, 34-36.

Rozen, S., Skaletsky, H., 2000. Primer3 on the WWW for general users and for biologist programmers. Methods Mol. Biol. 132, 365-386.
