# Supplementary material for: Expression patterns of five polymorphic membrane proteins during the Chlamydia abortus developmental cycle
Source: Vet Microbiol. 2012 Dec 7;160(3-4):525–9. doi: 10.1016/j.vetmic.2012.06.017 (PMC3504296; doi:10.1016/j.vetmic.2012.06.017)
Supplement: Supplementary file 2 [file mmc2.docx]

**Supplementary results**

Results of antibody validation

*Antibody immuno-reativity and cross-reactivity*

PCR-amplification of each of the *pmp* genes provided amplified products of the expected size. Each of the products was successfully cloned into the expression vector in the correct orientation as demonstrated by restriction enzyme digestion (data not shown). All passenger domain constructs were successfully expressed as single recombinant proteins except for Pmps 1B, 15G and 18D. The passenger domains for each of these 3 proteins were expressed as either 2 (Pmp1B, 15G), or 3 overlapping fragments (Pmp18D) (Supplementary Table). Each of the recombinant proteins were found to be expressed in the insoluble fraction. After SDS-PAGE, bands corresponding to each expressed protein were successfully identified by MALDI-ToF analysis, with subsequent identification of multiple specific peptide matches by BLASTp against the NCBI non-redundant protein database.

Each of the antibodies reacted with each of the relevant expressed Pmp constructs (Figure 1). A number of additional reactive bands of lower than expected molecular weight were identified by each antibody. Analysis of some of these bands by MALDI-TOF identified peptide sequences corresponding to each of the specific constructs and perhaps indicates products of protein degradation or processing. The specificities of each of the antibodies targeting unique peptides in Pmp10G, Pmp13G, Pmp16G, Pmp17G and Pmp18D were determined and tested for cross reactivity against each of the remaining recombinant Pmps. Each polyclonal antibody was specific for the relevant Pmp recombinant protein with no cross-reactivity observed (Supplementary Figure 1). In addition no specific reactivity against any of the Pmp constructs could be observed after incubation with pre-immune rabbit serum.
