# Supplementary material for: Expression patterns of five polymorphic membrane proteins during the Chlamydia abortus developmental cycle
Source: Vet Microbiol. 2012 Dec 7;160(3-4):525–9. doi: 10.1016/j.vetmic.2012.06.017 (PMC3504296; doi:10.1016/j.vetmic.2012.06.017)

Supplementary figure 1. Immunoblot of recombinant Pmps with anti-Pmp antibodies. Recombinant proteins were separated by SDS-PAGE and Coomassie blue-stained, or immunoblotted with anti-Pmp antibodies as indicated. Asterisks indicate specific reactivity against individual Pmps. §Where Pmp passenger domains were expressed as multiple overlapping constructs these are donated by a b c

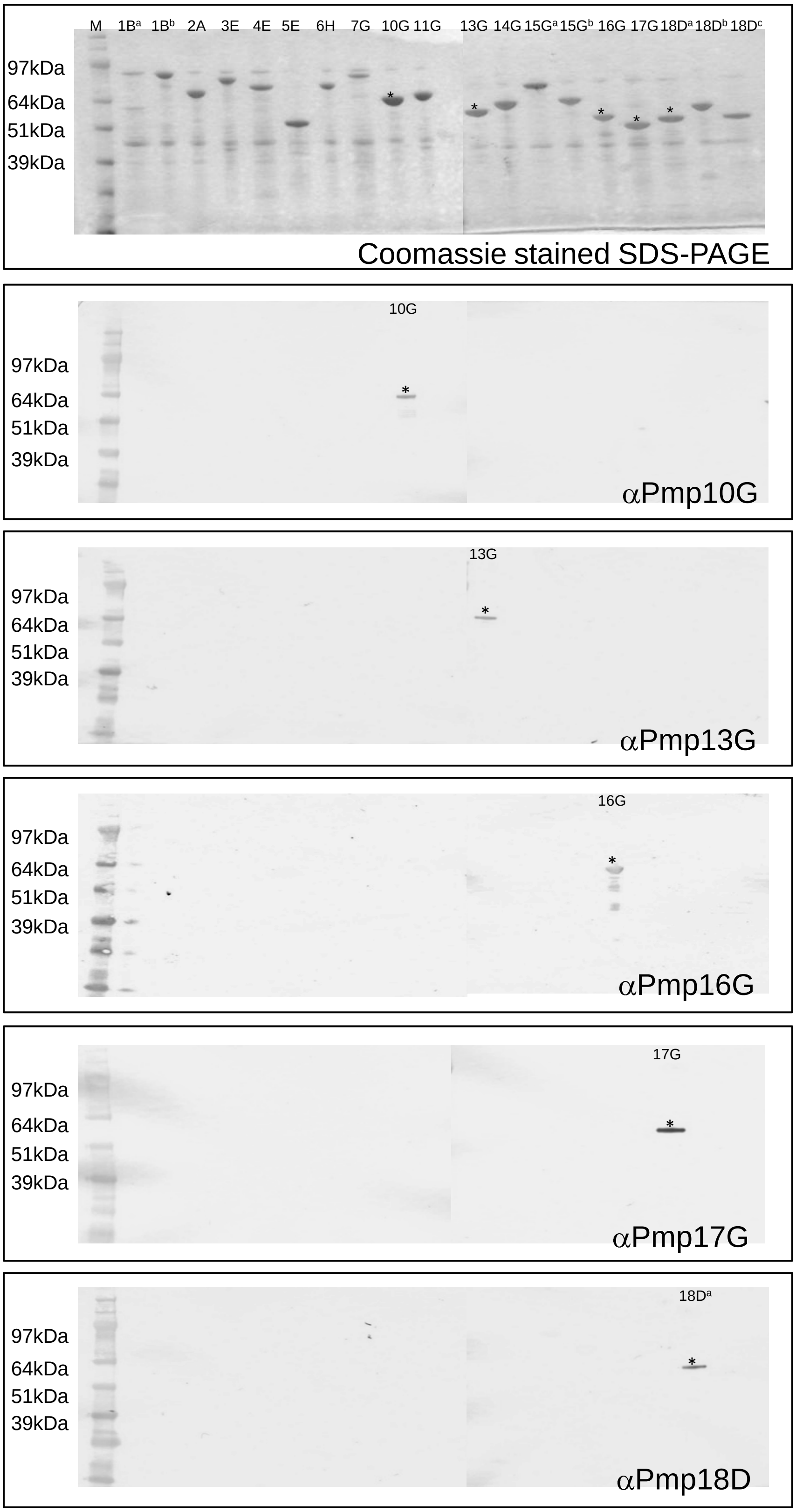

Supplementary Figure 2. Fluorescent micrographs demonstrating the expression of Pmps at 60h pi. Fluorescent immunocytochemistry was carried out as described (Materials & Methods). Slides were incubated with α-Pmp and α-OMP-1 antibodies and visualized with α-rabbit-FITC (Green, panels A-E) and α-mouse-Alexafluor 598 (Red, panels, F-J) antibodies respectively. Combined images are also shown (panels K-O). Pmp10G (A, F, K); Pmp13G (B, G, L); Pmp16G (C, H, M); Pmp17G (D, I, N); and Pmp18D (E, J, O). All micrographs were obtained at the same magnification, and a scale bar is highlighted in panel O

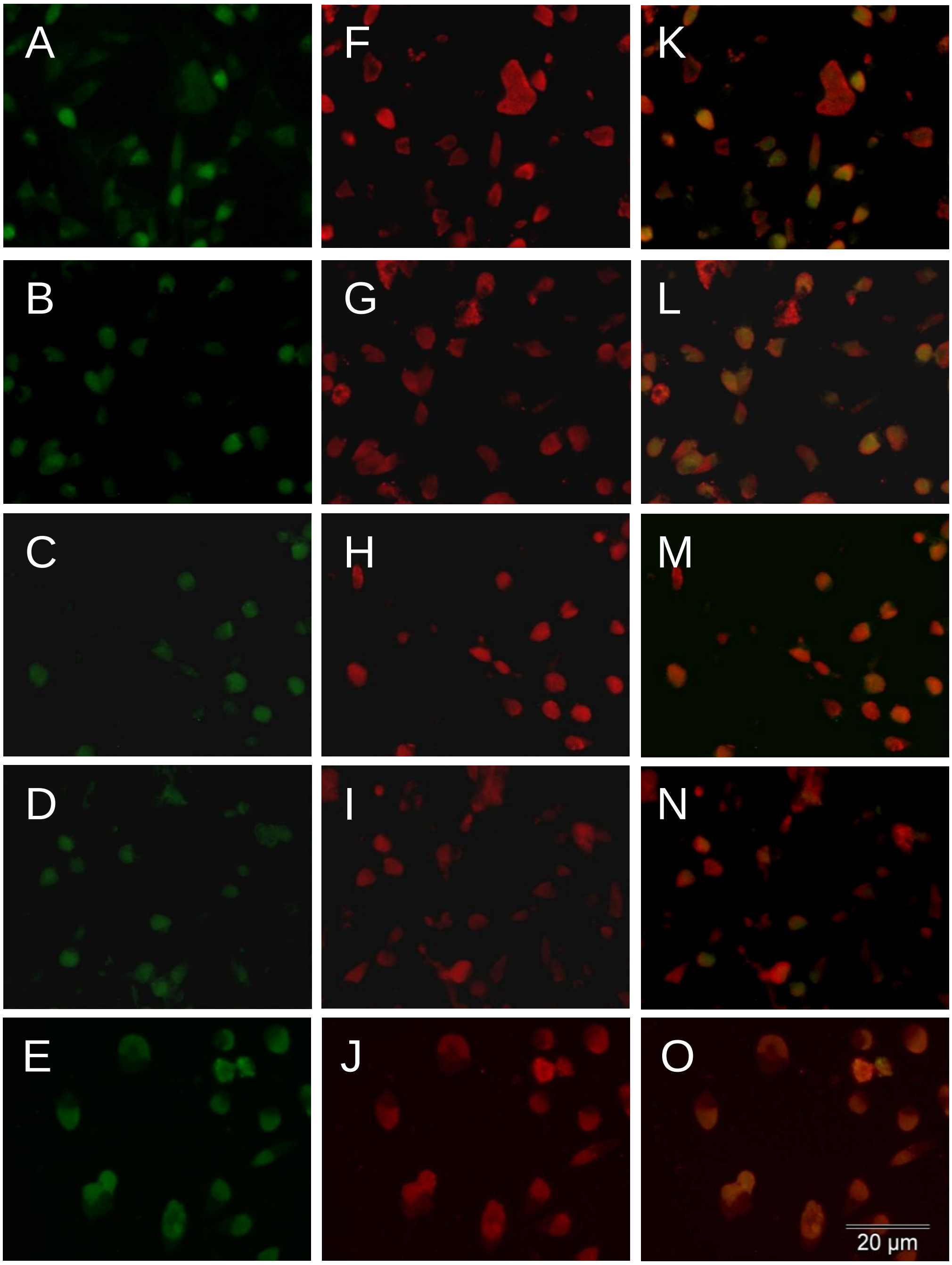

Supplement: Supplementary file 3 [file mmc3.pdf]
