# Supplementary material for: Expression patterns of five polymorphic membrane proteins during the Chlamydia abortus developmental cycle
Source: Vet Microbiol. 2012 Dec 7;160(3-4):525–9. doi: 10.1016/j.vetmic.2012.06.017 (PMC3504296; doi:10.1016/j.vetmic.2012.06.017)
Supplement: Supplementary file 4 [file mmc4.docx]

Supplementary Table 1. Primers used to PCR-amplify Pmp passenger domains used for generating recombinant proteins.

| Gene^§^ | Forward primer (5’-3’)* | Reverse primer (5’-3’) | Amino acids^#^ |
| --- | --- | --- | --- |
| *pmp1B* a  b | CACCATGGAACCTCTTTCGAAAGAATT  CACCATGGTGATCACTGGCAACAAAGC | TCCTATTGCTGCAACACCTG  GATATAAATCCAGCGGCGGTA | 22-770  717-1460 |
| *pmp2A* | CACCATGCCACTGACTCAATTATCC | AGGGAACCAAAGAACCTTCC | 17-631 |
| *pmp3E* | CACCATGGAAGCCTATCTAAAAGATATA | ATTAGCAATCAGCGGGGTCA | 26-689 |
| *pmp4E* | CACCATGTACGAGCTCATTTGCCTT | ATTAGCCACAAGATCTCCACGA | 19-646 |
| *pmp5E* | CACCATGAAATCCACAATTACAAAAT | TTTGCCCCCAGCAAGTAGAT | 1-356 |
| *pmp6H* | CACCATGCAAACTAACCTTACACCTCCTTTG | ATGTGTGGGTTCTGGTGTCC | 29-650 |
| *pmp7G* | CACCATGTTGGAAATTGTAGTTCCT | ATTAGGACTAAAGAAGCTTGACG | 25-722 |
| *pmp10G* | CACCATGGAAAACTTAGGTTCTTC | AGGAACTAAAGGTGTAGTGATTTCTT | 24-517 |
| *pmp11G* | CACCATGGCTACCAAACTTCTTGCA | AGGCACTAAAGATGTCGTAAACTC | 24-527 |
| *pmp13G* | CACCATGGAGGAACCAGATCA | CGGAACTAAAGATCCTTGACG | 23-524 |
| *pmp14G* | CACCATGACTGCGGGAGGAACGAT | GAGTACTAGCGGAGCACGAC | 50-606 |
| *pmp15G* a  b | CACCCATGACAGGGGAACAAACACTAA  CACCATGGTCTTCTCTGGCAACCAAGC | TCCCGAGGAGTTATTGGAGA  ATTAGGGACGAGCGTAGCACGT | 35-598  471-1050 |
| *pmp16G* | CACCATGTCCTATGCAGGGAAAGAT | TGGATTAGGAGAGTAACCCGTTT | 65-515 |
| *pmp17G* | AGCATATGGCTAACGACGCTCAAACAG | GAATTCCTAAAGGTCCTTGACGTTCTGGGT | 23-513 |
| *pmp18D* a  b  c | CACCGGTTATGTACCAGCTTCAGAG  CACCATCGTAGAGAACGTTGGCAGTT  CACCAGAGCGATAATACAAAAGTCGTGA | AGAACTAATCGCACCACCAGTGAC  TTTTGTATTATCGCTCTCTACTTTAGC  CGATAGCCCCACTCTTCTCA | 37-418  411-840  835-1220 |

^§^Where Pmp passenger domains were expressed as multiple overlapping constructs these are donated by ^a b c^

*Forward primers for cloning into pET102/D-TOPO^®^ have additional CACC at 5’ terminus

# Amino acid positions in relation to N-terminus of the gene product (excluding predicted signal peptide sequences where present)
